# Steady-state visual evoked potentials in children with neurofibromatosis type 1: associations with behavioral rating scales and impact of psychostimulant medication

**DOI:** 10.1186/s11689-022-09452-y

**Published:** 2022-07-22

**Authors:** Eve Lalancette, Audrey-Rose Charlebois-Poirier, Kristian Agbogba, Inga Sophia Knoth, Emily J. H. Jones, Luke Mason, Sébastien Perreault, Sarah Lippé

**Affiliations:** 1 Department of Psychology, University of Montreal, Marie Victorin Building, 90 Vincent-D’Indy Avenue, Montreal, Quebec H2V 2S9 Canada; 2 CHU Sainte-Justine Research Center, 3175 Côte Ste-Catherine, Montreal, QC H3T 1C5 Canada; 3 Centre for Brain and Cognitive Development, Birkbeck, University of London, 32 Torrington Square, London, WC1E 7JL UK; 4 Department of Neurosciences, Division of Child Neurology, CHU Sainte-Justine, 3175 Côte Ste-Catherine, Montreal, QC H3T 1C5 Canada

**Keywords:** Neurofibromatosis type 1, Steady-state visual evoked potentials, Electroencephalography, Inattention symptoms, Psychostimulant medication

## Abstract

**Background:**

Neurofibromatosis type 1 (NF1) is a genetic disorder often associated with cognitive dysfunctions, including a high occurrence of deficits in visuoperceptual skills. The neural underpinnings of these visuoperceptual deficits are not fully understood. We used steady-state visual evoked potentials (SSVEPs) to investigate possible alterations in the synchronization of neural activity in the occipital cortex of children with NF1.

**Methods:**

SSVEPs were measured using electroencephalography and compared between children with NF1 (*n* = 28) and neurotypical controls (*n* = 28) aged between 4 and 13 years old. SSVEPs were recorded during visual stimulation with coloured icons flickering at three different frequencies (6 Hz, 10 Hz, and 15 Hz) and analyzed in terms of signal-to-noise ratios. A mixed design ANCOVA was performed to compare SSVEP responses between groups at the three stimulation frequencies. Pearson’s correlations with levels of intellectual functioning as well as with symptoms of ADHD, ASD and emotional/behavioral problems were performed. The impact of psychostimulant medication on the SSVEP responses was analyzed in a subset of the NF1 group (*n* = 8) with paired t-tests.

**Results:**

We observed reduced signal-to-noise ratios of the SSVEP responses in children with NF1. The SSVEP responses were negatively correlated with symptoms of inattention and with symptoms of emotional/behavioral problems in the NF1 group. The SSVEP response generated by the lowest stimulation frequency (i.e., 6 Hz) was rescued with the intake of psychostimulant medication.

**Conclusions:**

Impaired processing of rhythmic visual stimulation was evidenced in children with NF1 through measures of SSVEP responses. Those responses seem to be more reduced in children with NF1 who exhibit more symptoms of inattention and emotional/behavioral problems in their daily life. SSVEPs are potentially sensitive electrophysiological markers that could be included in future studies investigating the impact of medication on brain activity and cognitive functioning in children with NF1.

**Supplementary Information:**

The online version contains supplementary material available at 10.1186/s11689-022-09452-y.

## Background

Neurofibromatosis type 1 (NF1) is an autosomal dominant genetic disorder caused by pathogenic alterations in the NF1 gene. With an estimated prevalence of one in 3000, it is one of the most common inherited disorders affecting the human nervous system [1, 2]. The NF1 gene encodes neurofibromin, a protein that is expressed in various cell types, and especially in neurons and glial cells [3]. Neurofibromin acts as a negative regulator of Ras-mediating signaling pathways which control cell growth and proliferation [4]. While NF1 affects multiple systems of the body, the most frequent complications experienced in childhood are cognitive dysfunction and behavioral problems [5]. Although the causes of these complications are not fully understood, studies on animal models have helped elucidate how neurotransmitter imbalances related to disruptions in Ras molecular pathways contribute to the NF1 cognitive and behavioral phenotype. In mouse models of NF1, disrupted γ-aminobutyric acid (GABA) and dopamine neurotransmission have been shown to be implicated in learning, memory, and attention system deficits [6–8], and are thought to contribute to neurodevelopemental disorder risk in NF1 [9]. However, how these perturbations in GABAergic and dopaminergic neurotransmission translate into altered neural activity in NF1 should be investigated to further our understanding of the cognitive deficits and develop effective biomarkers in this population.

Children with NF1 are at increased risk of developing several neurodevelopmental problems. Around 20% of children with NF1 meet diagnostic criteria for specific learning disorders [10], but up to 75% perform more than one standard deviation below their grade peers in reading, writing, or mathematics-related skills [11]. They are therefore much more likely to receive special education or remedial teaching throughout their schooling [11]. The internalizing and externalizing behavior problems as well as the social difficulties experienced by children with NF1 can also impact their development and quality of life [12–14]. In some cases, social difficulties are related to a comorbid diagnosis of autism spectrum disorder (ASD), the incidence of which is estimated at 25% of children with NF1 [15–17]. Inattention and hyperactivity/impulsivity symptoms consistent with the attention-deficit/hyperactivity disorder (ADHD) diagnosis are very common as well. Whether these symptoms result from ADHD as an independent comorbidity or are a consequence of NF1 is still a matter of debate [18]. Nevertheless, between 38 and 67% of children with NF1 meet diagnostic criteria for ADHD [19, 20] and the comorbidity was shown to have a negative impact on the intellectual outcome and academic achievement [18, 21]. As a result, a large proportion of children with NF1 are treated with methylphenidate, a psychostimulant medication often prescribed for ADHD due to its action on the dopaminergic and noradrenergic systems [22], or with other psychostimulant medications. Methylphenidate is thought to have beneficial effects on the cognitive performance of children with NF1 [23, 24], but the impact on measures of brain activity is still unknown.

Regarding specific domains of cognitive functioning, visuoperceptual deficits have been particularly studied and are considered a robust characteristic of the NF1 cognitive phenotype. Lower performances have been consistently found on the Judgement Orientation Line test [25], a standardized test of visuospatial perception measuring the ability to match the angle and orientation of lines in space [26]. Deficits in the perceptual analysis of shapes and their spatial features [27], impairments in visual learning [28, 29] and abnormal reactivity to visual signals [30] have also been evidenced using various cognitive tasks. Moreover, children with NF1 exhibit reduced visuomotor integration [31] and a high prevalence of motor problems [32].

Structural and functional brain abnormalities evidenced in patients with NF1 have helped to understand the neurobiological basis of visuoperceptual deficits. In a functional magnetic resonance imaging (fMRI) study, Clements-Stephens, et al. [33] reported inefficient recruitment of the right hemisphere network and hypoactivation of the primary visual cortex in children with NF1 while performing the Judgment of Line Orientation task. Violante et al. [34] also found reduced activation of the low-level visual cortex in both children and adults with NF1 during magnocellular and parvocellular stimulations. Moreover, the authors found a deficient deactivation of the default mode network in response to magnocellular stimulation, which was hypothesized to be related to the attentional deficits in this population. In an EEG study, Ribeiro et al. [35] reported anomalies in the long-latency components of the visually-evoked potential in response to chromatic stimulation. They also found abnormal enhancement of alpha oscillations in the parieto-occipital cortex, which was related to increased attentional lapses in a visual detection task. A better understanding of the differences in the functioning of the occipital cortex in NF1 patients may therefore be useful in elucidating the visuoperceptual and attentional deficits in this population.

Electroencephalography (EEG) allows the investigation of visually evoked activity in a rapid and non-invasive way that is well suited for children. The periodic delivery of visual stimuli elicits a periodic neural response in the visual cortex, also known as steady-state visual evoked potentials (SSVEPs) [36]. SSVEPs appear in the EEG signal as a clear peak (or power increase) at the stimulation frequency and its harmonics [36, 37]. Since SSVEP responses have high signal-to-noise ratios (SNRs) and are robust to artifacts [38], they are particularly useful to study cerebral activity in young children [39]. SSVEPs can be recorded in various ways (e.g., magnetoencephalography (MEG), positron emission tomography (PET), fMRI), but EEG remains the method of choice due to its precise temporal resolution and accessibility [40]. The generating mechanism of the steady-state response is a matter of debate, especially concerning its relationship with spontaneous (or endogenous) oscillatory activity [41–43]. Nevertheless, it is thought to emerge from the synchronization of neural activity to the stimulus frequency via phase alignment [44]. SSVEP cortical sources are found mainly in the primary visual cortex, but depending on the frequency of stimulation, contributions have also been identified from the frontal cortex and from extracortical structures [40]. Additionally [38, 39], maturational changes of the SSVEP response occur during childhood. For example, increases in magnitude values of the 5 Hz SSVEP were seen until 8–11 years old in the occipital region, while phase alignment values reached their maximum in adulthood [45]. Considering the altered trajectory of brain development evidenced in NF1 [46, 47], those maturational changes could be delayed in children with NF1, thus reducing the overall magnitude of the SSVEP response.

Very limited studies have investigated SSVEPs in children with NF1. One study assessed the SSVEP response to the stimulation of the central and peripheral visual fields (at 8 Hz and 7.2 Hz, respectively) in a sample of 10 children with optic pathway gliomas, 7 of which were diagnosed with NF1, and compared to 33 healthy controls (ages 3 to 18 years). Results showed lower SNR of the SSVEP response in children with optic pathway gliomas in response to the central stimulation, but no group difference was observed in the peripheral visual field [48]. Furthermore, in their study of visual evoked potentials and brain oscillations in a sample of 17 NF1 participants and 18 controls (ages 8 to 17 years), Ribeiro, et al. [35] reported higher amplitude of non-phase-locked alpha oscillations elicited by a 5-Hz achromatic stimulation, but did not specifically analyze the amplitude of the SSVEP response in terms of SNR. These results indicate the need for further investigation of the SSVEP response in children with NF1 using larger sample sizes and across a wider range of stimulation frequencies.

Atypical steady-state responses have been found in various psychiatric disorders, including schizophrenia [49, 50] and depression [51], as well as in other neurodevelopmental disorders such as ADHD [52] and ASD [53]. It is therefore relevant to investigate whether the possible SSVEP alterations in NF1 could be associated with behavioral symptoms of comorbid neurodevelopmental disorders or emotional problems. Psychostimulant medication, such as methylphenidate and amphetamines, could have an impact on the SSVEP response. Functional imaging studies revealed that such medication strengthens the connectivity between the dorsal attention network and the thalamus, as well as between the thalamus and sensory regions including the visual cortex, which suggests a modulation of sensory processing by psychostimulants [54]. In a study examining the effects of methylphenidate on various electrophysiological markers of sustained attention in healthy adults, Dockree, et al. [55] found no significant effect on the SSVEP amplitude generated by a patterned stimulus flickering at a rate of 25 Hz. However, the effect of psychostimulant medication on SSVEPs generated by lower frequencies is still unknown, and so is the effect of psychostimulants on the SSVEP amplitude in children with ADHD or NF1. Other EEG correlates of methylphenidate’s impact on brain activity have been identified in children with ADHD, such as normalization of the theta/beta ratio [56, 57] and of the P300 event-related potential component [58, 59]. Whether children with NF1-ADHD differ from general ADHD samples in this regard has yet to be examined [9].

In this study, we investigated the synchronization of occipital activity in children with NF1 and neurotypical controls aged between 4 and 13 years old by measuring the SSVEP response to the rhythmic delivery of visual stimuli at different frequencies. We also explored possible relationships between SSVEP and measures of intellectual and behavioral functioning. Moreover, we looked at the impact of psychostimulant medication on the SSVEP amplitude in a subset of NF1 participants.

## Objective and hypothesis

Our main goal was to evaluate the integrity of the SSVEP response in children with NF1. The SSVEP response may reflect the underlying molecular mechanisms that are altered in NF1 and would likely be correlated with higher level cognitive functions, such as attention. This cortical response might therefore be a relevant marker to evaluate the effectiveness of treatments aimed at improving cognitive functioning in NF1.

First, we compared the SSVEP response between children with NF1 and typically developing children at three stimulation frequencies (6 Hz, 10 Hz, 15 Hz). We hypothesized that children with NF1 would show reduced SSVEP amplitude at all frequencies.

Secondly, we explored the associations between the SSVEP response and measures of intellectual and behavioral functioning. At the behavioral level, we looked more specifically at symptoms of ADHD, ASD, and emotional problems. We hypothesized that lower SSVEP amplitude would be associated with increased symptomatology*.*

Thirdly, we investigated the impact of psychostimulant medication intake on the SSVEP response with the hypothesis that medication would increase SSVEP amplitude and thus normalize the EEG signal.

## Methods

### Participants

Thirty-one children with NF1 were recruited in collaboration with the Neurofibromatosis clinic at CHU Sainte-Justine, all of which met the revised diagnostic criteria for NF1 from the International Consensus Group on Neurofibromatosis Diagnostic Criteria [60]. Children with a history of neurosurgery or taking anticonvulsants were excluded. NF1 participants taking psychostimulant medication were recruited and offered to come back to the laboratory for a second EEG without medication (after a 24-h washout), within 1 month following the first visit. Three participants did not perform this second EEG without medication and were thus excluded from the analyses. As a result, 28 children with NF1, ages 4 to 13 years old, were included in the EEG analyses.

Comorbidities and medication in the NF1 group are listed in Table 1. Among the 14 participants with the NF1-ADHD comorbidity, 8 were taking psychostimulant medication and were tested with and without medication, with a mean interval of 22 days (SD = 6) between both EEG recordings. Three NF1 participants presented with optic pathway gliomas, identified by an ophthalmological evaluation. Non-parametric tests were performed to ensure that the EEG measures of these participants were not significantly different from the rest of the NF1 group and could be included in the analyses (see Additional file 1).

**Table 1:** Comorbidities and medication in the NF1 group

	NF1 group
***N***	28
**Comorbidities**	
History of seizures	1
Intellectual disability	2
Tourette’s syndrome	1
ASD	1
ADHD	14
**Medication**	
Methylphenidate	4
Lisdexamfetamine	3
Amphetamine	1
Dosage (mg) *Mean (SD)*	35 (12.09)
Time between the two EEG recordings (days) *Mean (SD)*	22 (6)

*Abbreviations*: *NF1* neurofibromatosis type 1, *ASD* autism spectrum disorder, *ADHD* attention-deficit/hyperactivity disorder, *mg* milligram, *EEG* electroencephalography, *SD* standard deviation

Twenty-eight controls, also aged from 4 to 13 years old, were recruited through social media adds and posters in public libraries. Exclusion criteria included any neurological condition, psychological or developmental disorder and intake of medication.

Demographic, cognitive and behavioral characteristics of both groups are presented in Table 2. No significant differences were found between groups in terms of age and sex ratios, as well as in household income. As previously demonstrated [61], the NF1 group’s mean IQ scores was slightly below average and was significantly different from the control group’s mean IQ which was in the upper limit of the average range. Behavioral symptoms captured by parental questionnaires were significantly higher in the NF1 group for all selected scales and total scores. In the NF1 group, mean scores on the Conners 3 Inattention scale and Global Index fell above clinically significant cut-offs (above high average; *T* scores ≥ 63). As for the SRS-2 Total score and CBCL Total problems, NF1 participants’ mean scores fell in the high average range (*T* scores between 57 and 63).

**Table 2:** Demographic, cognitive, and behavioral characteristics of the NF1 and control groups

	NF1 group (***N*** = 28)	Control group (***N*** = 28)	Group comparison	
	*Mean (SD or %)*	*Mean (SD or %)*	*T test or χ*^*2*^	*p*
Age (years)	9.39 (2.41)	8.88 (2.41)	0.80	0.43
Sex (*N* females)	15 (53.57%)	13 (46.43%)	0.29	0.59
Household income ($CAN)	85 608.70 (32 610.29)	124 652.17 (96 939.35)	− 1.83	0.08
IQ	89.00 (10.96)	108.61 (13.32)	− 6.02	0.0000002
Conners 3 Inattention Scale	68.23 (15.42)	53.52 (10.87)	3.89	0.0003
Conners 3 Global Index	65.31 (17.91)	53.43 (10.44)	2.83	0.007
SRS-2 total score	58.50 (11.80)	48.39 (7.12)	3.88	0.0003
CBCL total problems	57.32 (11.23)	47.30 (9.92)	3.50	0.001

*Note*: IQ results are presented in standard scores. Results from the Conners 3, the SRS-2 and the CBCL questionnaires are presented in *T*-scores, with higher scores representing more symptoms

*Abbreviations*: *NF1* neurofibromatosis type 1, *SD* standard deviation, *IQ* intellectual quotient, *Conners 3* Conners 3rd edition, *SRS-2* Social Responsiveness Scale 2nd edition, *CBCL* Child Behavior Checklist

The study was approved by the hospital’s research ethics board. All participants’ parents provided written informed consent to participate and were free to withdraw at any point. Children also gave their verbal or written consent after receiving explanations of the study’s purpose and procedures which were adapted to their level of understanding

### Experimental protocol

#### Neuropsychological evaluation and parental questionnaires

Participants’ intellectual functioning was assessed during a neuropsychological evaluation conducted by a graduate student in Clinical Neuropsychology. The Wechsler Preschool and Primary Scale of Intelligence, Fourth Edition (WPPSI-IV) was administered for children between 4 and 5 years old and the Wechsler Intelligence Scale for Children, Fifth Edition (WISC-V) for those between 6 and 13 years old. The subtests were administered in the recommended order and breaks were scheduled during the session. NF1 participants taking psychostimulant medication did the neuropsychological evaluation with medication on their first visit.

Parents completed questionnaires prior to or during the testing in order to assess behavioral symptoms of ADHD, ASD, and emotional problems. ADHD-related behaviors were assessed using the Conners 3rd Edition–Parent (Conners 3-P), ASD-related behaviors using the Social Responsiveness Scale, 2nd Edition (SRS-2) and emotional/behavioral problems using the Child Behavior Checklist (CBCL).

#### EEG acquisition

EEG recordings were performed in a dark, electrically shielded and soundproof room at CHU Sainte-Justine, using 128 electrode nets (Electrical Geodesics System Inc., Eugene, OR, USA). Signals were acquired and stored in a G4 Macintosh computer using NetStation EEG Software (Version 4.5.4) and sampled at 1000 Hz. During recording, impedances were kept under 40 kΩ and Cz acted as the reference electrode. Visual stimuli were presented on a Tobii T120 Eye Tracking screen with 1024 × 1280-pixel resolution and a refresh rate at 60 Hz. The screen was located at 60 cm from the participants’ eyes and subtended a visual angle of 31° in width and 25° in height. Children were instructed to remain calm, to limit movements, and to look at the screen throughout the task. An eye-tracking device (Tobii T120) was used to monitor the children’s gaze during the expriment. A research assistant remained in the room with the participants and reminded them to look at the stimuli if the eye-tracking indicated that their gaze left the screen.

#### Visual task

**Fig. 1:**
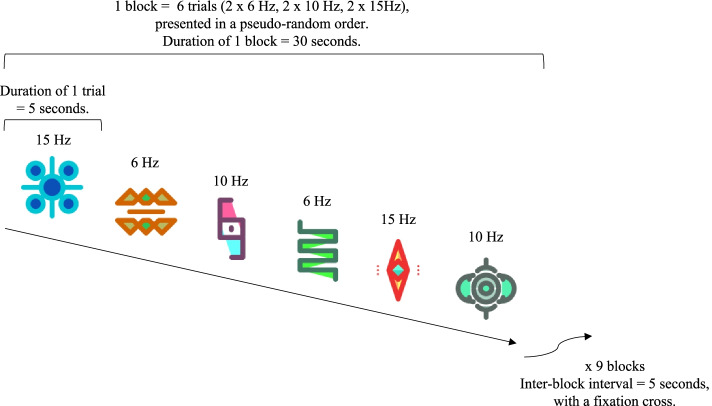
Design of the visual stimulation task

Visual stimuli consisted of 18 colored icons (see Additional file 2) appearing and disappearing (onset/offset pattern) at the center of the screen at a frequency of 6 Hz, 10 Hz, or 15 Hz. Coloured icons subtented a mean visual angle of 12.68° in height. Luminance has been normalized between the different icons and the mean luminance of the stimuli was 121.84 cd/m^2^. Stimuli were presented via E-Prime 2.0 (Psychology Software Tools Inc. Pittsburgh, PA, USA). Each 5 s trial displayed one icon at one frequency. Each block was composed of 6 trials (2 at 6 Hz, 2 at 10 Hz, 2 at 15 Hz) presented in a pseudo-random order (see Fig. 1). The task contained 9 blocks, with 5 s pause in between blocks showing a fixation cross, for a total duration of 5 min and 10 s. The visual stimuli were designed to be attractive and to enhance young children’s attention during the task.

### Analyses

#### Off-line EEG processing

Off-line signal processing and analyses were carried out using MATLAB R2018b (Mathworks, Inc., Natick, MA) and the EEGLAB toolbox v14 [62]. A 0.5–150 Hz bandpass filter and a notch at 60 Hz were applied. Twenty-eight electrodes located around the neck and containing muscular artifacts were removed. Remaining noisy electrodes were removed using a semi-automatic procedure. First, electrodes with a standard deviation lower than 2 μV or higher than 200 μV were automatically excluded. Secondly, a visual inspection was performed and electrodes with sporadic behavior were manually removed. Data was re-referenced to an average reference. Eye movement artifacts (blinks and saccades) and cardiac artifacts were removed with a semi-automatic independent component analysis (ICA, runica algorithm), as implemented in the EEGLAB toolbox [62]. Components were visually inspected and manually rejected if they showed prototypical patterns of blinks, saccades or cardiac activity. Data was then segmented into 5 s epochs (from the onset of the stimulation to 5 s post-onset) that were visually inspected and rejected if containing significant artifacts. All participants met the requirement of having a minimum of 6 remaining epochs (30 s) in each condition after artifact rejection. The average number of ICA components rejected and the average number of artifact-free epochs kept in each stimulation frequency are shown in Tables 3 and 4. No significant difference was found in these pre-processing indicators compared between the NF1 and control groups. Pre-processing indicators of NF1 participants who performed EEG recordings with and without medication could not be statistically compared given the small sample size, but means and standard deviations are presented in Table 4.

**Table 3:** EEG pre-processing indicators and groups comparison between the NF1 and control groups

	NF1 (***N*** = 28)	Controls (***N*** = 28)	Group comparison	
	*Mean (SD)*	*Mean (SD)*	*T test*	*p*
**ICA components rejected**	2.18 (1.19)	2.00 (0.86)	0.64	0.52
**Artifact-free epochs**				
**6 Hz**	13.68 (2.72)	12.89 (3.36)	0.96	0.34
**10 Hz**	13.43 (3.04)	12.89 (3.19)	0.64	0.52
**15 Hz**	13.29 (2.92)	12.75 (2.88)	0.69	0.49

*Abbreviations*: *NF1* neurofibromatosis type 1, *SD* standard deviation, *ICA* independent component analysis, *Hz* hertz

**Table 4:** EEG pre-processing indicators in the subset of NF1 participants tested with and without medication

	NF1 with medication (***N*** = 8)	NF1 without medication (***N*** = 8)
	*Mean (SD)*	*Mean (SD)*
**ICA components rejected**	2.38 (0.52)	2.00 (0.54)
**Artifact-free epochs**		
**6 Hz**	12.25 (2.05)	11.38 (1.69)
**10 Hz**	11.63 (3.25)	10.63 (2.34)
**15 Hz**	12.25 (2.49)	10.88 (2.17)

*Abbreviations*: *NF1* neurofibromatosis type 1, *SD* standard deviation, *ICA* independent component analysis, *Hz* hertz

#### SSVEP analysis

Fast Fourier transforms (FFTs) were performed on each of the 5-s epochs and averaged for each stimulation frequency (6, 10, and 15 Hz). The resulting power spectrum had a frequency resolution of 0.5 Hz. SSVEP amplitude was compared between groups in terms of SNRs. To obtain SNRs, we calculated the ratio between the amplitude at the stimulation frequency and the mean amplitude of both adjacent frequencies in a 1-Hz range, and then applied a logarithmic transformation [36, 39, 40].

Our region of interest for SSVEP analysis consisted of seven electrodes in the Oz region (E70, E71, E74, E75, E76, E82, E83). Spectral amplitudes were calculated for each electrode and then averaged for statistical analyses. All participants had at least four remaining electrodes in the Oz region after pre-processing.

#### Behavioral measures

Behavioral symptoms recorded from the three parental questionnaires (Conners 3, SRS-2 and CBCL) were compared between groups and correlations with SSVEP responses were performed. For the SRS-2 and CBCL questionnaires, analyses were carried out on total scores. Specific scales from the questionnaires were not systematically investigated in order to limit the number of statistical tests and because their associations with SSVEP measures were not supported by specific hypotheses. Since the Conners 3 questionnaire does not include a total score, analyses were carried out on the Global Index which includes 10 items and is known as a sensitive measure of response to treatment as well as a measure of general psychopathology [63]. Given the known interaction between attentional processes and SSVEP amplitude [64], analyses were also performed on the Conners 3 Inattention scale.

#### Statistical analyses

Statical analyses were carried out using IBM SPSS, version 26 (IBM, Armonk, NY, USA). Normality of the distribution was verified using asymmetry and kurtosis values. Parametric tests were used since those values were all in acceptable ranges (i.e., asymmetry and kurtosis z-scores smaller than |1.96|), except for the comparison of EEG measures between participants with optic pathway gliomas and the rest of the NF1 group for which we used the non-parametric Mann-Whitney test (see Additional file 1). Homogeneity of variance was tested by Levene’s test.

*T* tests were performed to compare groups in terms of demographics, intellectual functioning, and behavioral symptoms. Sex ratios were compared using the Chi-squared test. SNRs of the SSVEP response were compared between groups at the three stimulation frequencies using a mixed design ANCOVA, with group (NF1, control) as a between-subjects factor and frequency (6 Hz, 10 Hz, 15 Hz) as a within-subjects factor. Knowing the effect of age on SSVEP amplitude [45], age was added in the analysis as a covariate. For the mixed ANCOVA, the assumption of homogeneity of regression slopes was verified and respected (i.e., no significant interaction between age and group). The assumption of sphericity was verified with Mauchly’s sphericity test and respected since the test was non-significant. Significant effects from the mixed ANCOVA were investigated using follow-up ANCOVAs and post hoc comparisons with Bonferroni corrections for multiple testing. Pearson’s correlations were performed to investigate whether SSVEP SNRs were associated with measures of intellectual functioning and behavioral symptoms. Given the large number of correlations performed and the exploratory nature of these analyses, false discovery rate (FDR) correction was applied to control for multiple comparisons in the correlational analyses [65]. Paired *t* tests were used to compare EEG measures in the conditions with and without medication. For this last analysis, we calculated the difference between the two conditions and verified the normality of the differences’ distribution [66].

## Results

### SSVEP responses

**Fig. 2:**
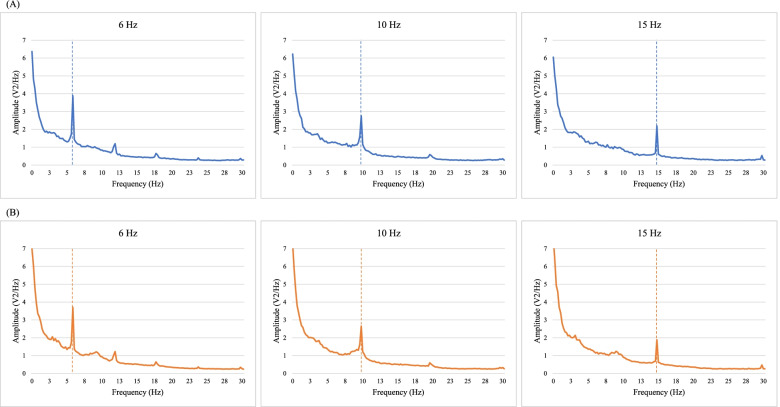
Averaged power spectrum resulting from the fast Fourier transforms in the control group (**A**) and NF1 group (**B**) for each stimulation frequency (6, 10, and 15 Hz). Dotted lines indicate the frequency of the visual stimulation at which the amplitude value was extracted.

Averaged power spectra resulting from the FFTs are shown in Fig. 2 for the NF1 and control groups at each visual stimulation frequency. Sharp peaks at the fundamental frequency (i.e., the stimulus frequency) and at its harmonics (i.e., multiples of the stimulus frequency) are evidenced in both groups. SSVEP responses were compared between groups with SNR measures, which take activity at the adjacent frequencies into account.

**Fig. 3:**
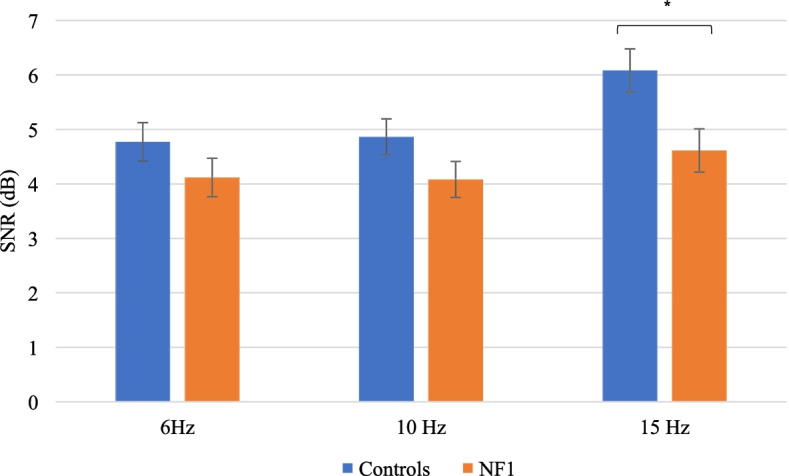
SSVEP SNR adjusted means (with standard errors as error bars) at each stimulation frequency by group. A main effect of group was found (*p* < 0.05) with lower SNRs in the NF1 group. Post hoc analyses in each stimulation frequency revealed a significant difference between groups for the 15 Hz stimulation. *p < 0.05

The mixed design ANCOVA confirmed that the covariate age was significantly related to the SSVEP SNRs (*F* (1, 53) = 22.24, *p* = 0.000018). After controlling for age, a main effect of group on SNR measures was found (*F* (2, 53) = 4.92, *p* = 0.031, partial η^2^ = 0.085), with higher SNR in controls (Fig. 3). The partial η^2^ indicates a medium to large effect size.

No significant interaction was found between groups and stimulation frequencies (*F* (2, 106) = 1.90, *p* = 0.16, partial η^2^ = 0.035). Post hoc ANCOVAs with Bonferonni adjustment were carried out to analyze the effect of group in each stimulation frequency individually. The covariate was significantly related to the SSVEP SNR in each stimulation frequency (*p* < 0.01). A significant difference between groups was found in the 15 Hz stimulation only (*F* (2, 53) = 6.78, *p* = 0.012, partial η^2^ = 0.11), with lower SNR in the NF1 group.

Given the high prevalence of NF1-ADHD comorbidity in our sample, we investigated how the inclusion of NF1 patients with a comorbid ADHD diagnosis (*N* = 14) could have affected our findings. To do so, we divided our cohort into three groups (controls, NF1 with ADHD, NF1 without ADHD) and performed our main analysis (mixed design ANCOVA). The main effect of group on SNR measures was still significant (*F* (2, 52) = 3.87, *p* = 0.027, partial η^2^ = 0.130) with a medium to large effect size. However, post hoc comparisons with Bonferroni adjustment revealed a significant difference solely between the control and NF1 with ADHD groups (*p* = 0.023) with higher SSVEP SNRs in controls. The NF1 without ADHD group did not differ from controls (*p > 0.05*), nor from NF1 participants with ADHD (*p > 0.05*).

### Relationship with IQ and behavioral symptoms

Exploratory correlational analyses were performed to identify possible relationships between SSVEP responses and measures of intellectual functioning or behavioral symptoms (see Table 5).

**Table 5:** Pearson correlations between SSVEP responses and measures of intellectual functioning and behavioral symptoms

	NF1 group			Control group		
	SNR at 6 Hz	SNR at 10 Hz	SNR at 15 Hz	SNR at 6 Hz	SNR at 10 Hz	SNR at 15 Hz
IQ	0.02	0.07	0.05	0.06	0.02	0.07
Conners 3 Inattention Scale	− 0.36	− 0.49^a,^*	− 0.55^a,**^	− 0.20	− 0.47*	− 0.33
Conners 3 Global Index	− 0.26	− 0.46 *	− 0.49^a,*^	− 0.03	− 0.22	− 0.06
SRS-2 Total Score	− 0.12	− 0.29	− 0.32	0.22	− 0.06	− 0.02
CBCL Total Problems	− 0.20	− 0.32	− 0.54^a,^**	0.33	0..18	0.17

*Abbreviations*: *NF1* neurofibromatosis type 1, *SNR* signal-to-noise ratio, *Hz* hertz, *Conners 3* Conners 3rd edition, *SRS-2* Social Responsiveness Scale 2nd edition, *CBCL* Child Behavior Checklist, *IQ* intellectual quotient

^a^Significant after false discovery rate (FDR) corrections. *p < 0.05. **p < 0.01. ***p < 0.001

**Fig. 4:**
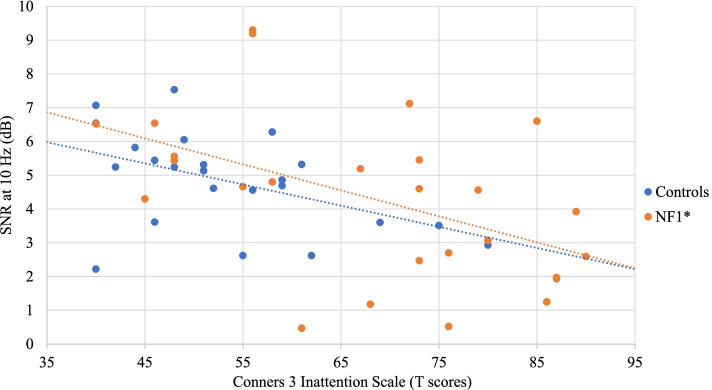
Relationship between the SSVEP SNR at 10 Hz and the Conners 3 Inattention scale . *p < 0.05; **p < 0.01

**Fig. 5.**
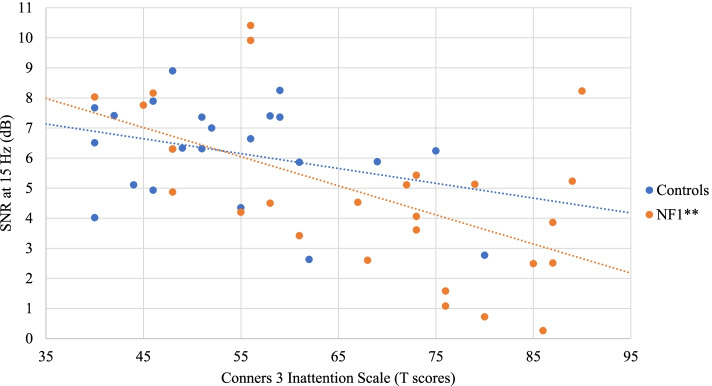
Relationship between the SSVEP SNR at 15 Hz and the Conners 3 Inattention scale. *p < 0.05; **p < 0.01

**Fig. 6:**
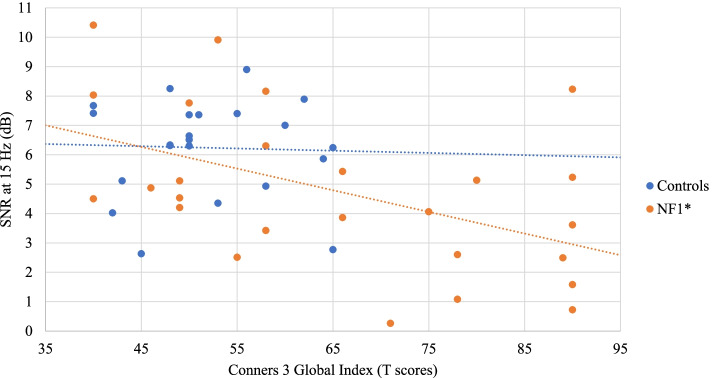
Relationship between the SSVEP SNR at 15 Hz and the Conners 3 Global Index. *p < 0.05; **p < 0.01

**Fig. 7:**
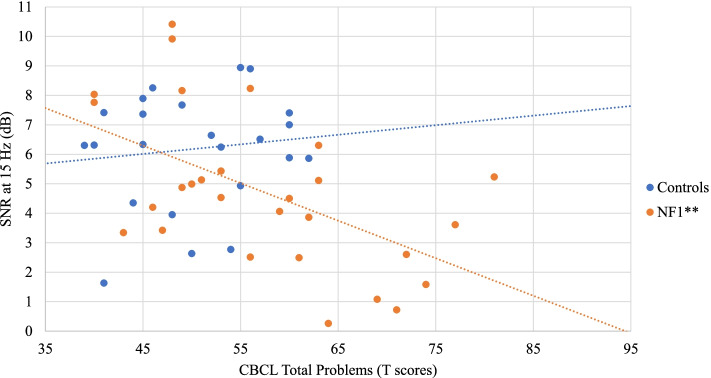
Relationship between the SSVEP SNR at 15 Hz and the CBCL Total problems scale. *p < 0.05; **p < 0.01

No significant correlation was found between SSVEP SNRs and IQ in both groups. As for associations with behavioral measures, no significant correlation (after FDR correction) was found in the control group. In the NF1 group, a negative correlation was found between SSVEP SNRs at 10 Hz and the Conners 3 Inattention scale (*r* = − 0.49, *p* = 0.011) (Fig. 4). Increased symptoms of inattention were therefore associated with reduced SNRs at 10 Hz. Similarly, SSVEP SNRs at 15 Hz were negatively correlated with the Conners 3 Inattention scale (*r* = − 0.55, *p* = 0.004) (Fig. 5), but also with the Conners 3 Global Index (*r* = − 0.49, *p* = 0.012) (Fig. 6). In addition, the SSVEP SNRs at 15 Hz had a negative relationship with the CBCL total problems (*r* = − 0.54, *p* = 0.003) (Fig. 7). These correlations remained significant after FDR correction.

### Impact of psychostimulant medication

**Fig. 8:**
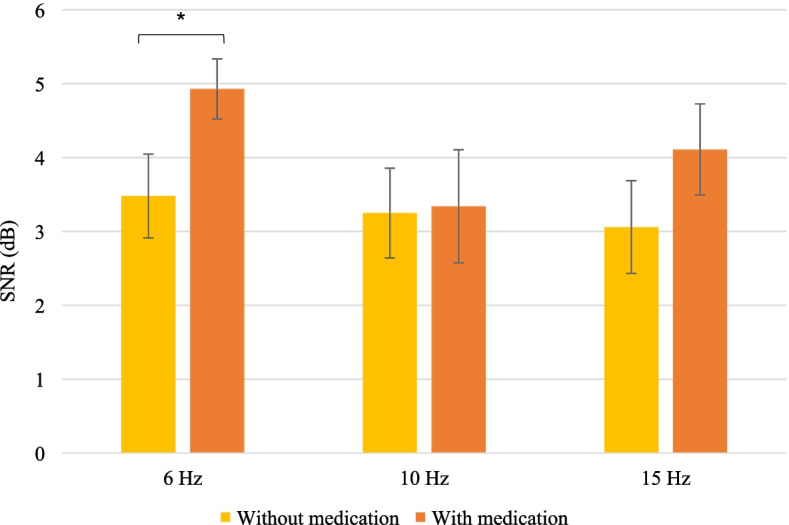
Mean SSVEP SNRs (with standard errors as error bars) in children with NF1 without and with psychostimulant medication (*N* = 8) at each stimulation frequency. *p < 0.05

Paired *t* tests were used to compare SSVEP responses in children with NF1 that are taking psychostimulant medication and who did the EEG recording with and without medication (*N* = 8). On average, children with NF1 showed higher SSVEP SNR at 6 Hz with psychostimulant medication (*M* = 4.97, SE = 0.41) than without medication (*M* = 3.48, SE = 0.57) (*t* (7) = 3.27, *p* = 0.014, *r* = 0.78). No significant impact of the psychostimulant medication intake was found for the 10 Hz (*t* (7) = − 0.07, *p* = 0.94, *r* = 0.03) and 15 Hz stimulations (*t* (7) = 1.68, *p* = 0.14, *r* = 0.54) (Fig. 8). The effect of medication on the SSVEP SNR at 6 Hz remains significant after Bonferroni adjustment for multiple comparisons which would set statistical significance at 0.017. However, this result should be interpreted with caution given the small sample size and the impossibility to control for age and number of epochs.

Since the impact of psychostimulant medication was investigated with a limited number of participants, the data was also analyzed in a descriptive manner, as shown in Table 6. For the 6Hz and 15 Hz stimulations, SNRs systematically increased with the intake of medication, except for one participant who presented multiple diagnoses (i.e., intellectual disability, ADHD, and Tourette’s syndrome). At 10 Hz, the impact of psychostimulant medication was rather uncertain, with three participants showing decreased SNRs after the intake of medication.

**Table 6:** Individual SSVEP SNRs from the EEG recordings without (left column) and with (right column) medication in eight participants with NF1

Participants	SNR at 6 Hz (dB)		SNR at 10 Hz (dB)		SNR at 15 Hz (dB)	
***N***	*Without medication*	*With medication*	*Without medication*	*With medication*	*Without medication*	*With medication*
**1**	2.94	4.65	3.07	1.38	0.72	2.01
**2**	4.88	3.73	3.92	1.62	5.23	3.69
**3**	4.61	6.52	2.70	1.14	1.58	2.23
**4**	5.08	6.37	4.80	4.93	4.50	4.93
**5**	4.52	6.02	1.18	1.53	2.60	3.27
**6**	0.36	3.93	0.52	2.92	1.08	6.02
**7**	2.72	4.06	4.66	5.76	4.20	4.38
**8**	2.71	4.47	5.19	6.41	4.53	6.95
**Mean**	3.48	4.97	3.25	3.21	3.05	4.18

*Abbreviations*: *SNR* signal-to-noise ratio, *Hz* hertz, *dB* decibels

## Discussion

The main objective of this study was to examine the SSVEP response in children with NF1 and neurotypical controls aged between 4 and 13 years old. We analyzed the SSVEP generated by coloured icons flickering at three different frequencies (6 Hz, 10 Hz, and 15 Hz). As exploratory objectives, we examined correlations with measures of intellectual functioning and behavioral symptoms, and investigated the impact of psychostimulant medication on the SSVEP response. Impairments in the generation of SSVEP could help elucidate neural mechanisms underlying visuoperceptual and attentional deficits in NF1 and eventually serve as a relevant marker of treatment efficacy [67].

Participants with NF1 showed clear peaks with maximal amplitude at the frequency of stimulation, but the SNRs of these peaks were decreased when compared to neurotypical controls. Moreover, our results show that the difference in SSVEP SNRs is increased in the subset of NF1 patients with comorbid ADHD. The SSVEP SNRs were not related to the level of intellectual functioning but were significantly correlated with behavioral symptoms captured by parental questionnaires. As such, SNRs were predominantly correlated with inattention symptoms measured by the Conners 3, but also with emotional and behavioral problems measured by the CBCL. Finally, an increase in the SNR at 6 Hz was seen with the intake of psychostimulant medication in participants with NF1.

### Reduced SSVEP responses in NF1

The significant main effect of group indicating reduced SSVEP in children with NF1 when compared to neurotypical controls confirmed our hypothesis. However, while we expected children with NF1 to show reduced SSVEP amplitude at all frequencies, separating the analyses by stimulation frequency showed significantly reduced SSVEP only for the 15 Hz stimulation. Reduced SSVEP responses in the NF1 group could result from structural and molecular abnormalities that have been identified in brain regions that underlie visual perception. In a MRI study of 39 participants with NF1 and 60 non-affected individuals, Duarte, et al. [68] used a multivariate data-driven classification approach to identify the most relevant brain regions that allowed discrimination between groups. Among those regions were the visual cortex and thalamus which showed characteristic structural differences. Thalamic hypometabolism has also been evidenced in children with NF1 [69]. While SSVEP originates in the primary visual cortex [40], the thalamus also plays an important role in visual processing by not only acting as a relay of visual information from the retina to the visual cortex, but also by influencing the spatial and temporal dynamics of the visual signal [70]. Thus, our results which show altered occipital response to rythmic visual stimulation are aligned with prior research in NF1, which indicated abnormal structure and function of brain regions that underlie visual processing. The reduced synchronization of occipital activity identified in our study could be associated with visuospatial processing deficits frequently reported in this population [71]. To clarify this link, future studies should investigate whether reduced SSVEP response can predict lower performance in cognitive tasks of visuospatial abilities in children with NF1.

At the molecular level, reduced SSVEP responses in NF1 could also reflect improperly balanced excitation and inhibition resulting from deficient GABA levels [72]. After having found reduced GABA levels in the visual cortex of children and adolescents with NF1 [73], Violante, et al. [72] showed that these deficits persist into adulthood, with lower concentration of GABA found in the occipital cortex and frontal eye fields. In addition, decreased binding of GABA_A_ receptors was found in the parieto-occipital cortex, midbrain, and thalamus of adults with NF1. GABA neuron-mediated inhibition plays an essential role in the synchronization of neural activity and generation of brain rhythms [74]. Deficient GABAergic neurotransmission in NF1 could therefore be associated with the impaired synchronization of occipital activity found in this study, which can provide new insight into to the emergence of visuoperceptual deficits in this population [71].

To further examine the reduced SSVEP response found in the NF1 group, we investigated how the inclusion of NF1 participants with an ADHD comorbidity could affect our findings. Half of the children part of the NF1 group has previously received a medical or neuropsychological diagnosis of ADHD. This proportion is in line with the prevalence generally reported in the literature, with rates ranging from 38 to 67% of children with NF1 meeting diagnostic criteria for ADHD [19, 20]. Our results showed that, when considering the ADHD comorbidity in NF1 participants, group differences in SSVEP responses are only found between the NF1 with ADHD subgroup and controls. This finding is consistent with the negative correlations found between the SSVEP amplitude and ADHD-related symptoms in the whole NF1 group.

A very limited number of studies have examined the steady-state response in children with ADHD. Khaleghi, et al. [52] investigated the auditory and visual steady-state response in adolescents with ADHD when performing a motor response inhibition task. In the visual modality, adolescents with ADHD showed higher SSVEP amplitudes at the prefrontal and frontal regions, but lower amplitudes at the temporal and occipital regions when compared to neurotypical controls. One way of interpreting their results was to suggest an abnormal connectivity between the anterior and posterior regions of the brain that could demonstrate deficits in the functional networks of frontoparietal and dorsal attention. While our experimental protocol did not require children to perform a specific task during the visual stimulation, our results are consistent with their findings with regards to the occipital region.

The ADHD comorbidity in children with NF1 has been shown to have an adverse influence on the cognitive profile [18] and adaptative functioning [75]. Further investigations are necessary to determine whether the greater difference on our electrophysiological measures is strictly attributable to the presence of an ADHD comorbidity in our NF1 participants or whether it is a consequence of more severe neurological deficits resulting from the NF1 mutation. However, given the high prevalence of ADHD in children with NF1, it is certainly relevant to include participants presenting this comorbidity in our analyses.

### Relationship with IQ and behavioral symptoms

The NF1 group’s mean level of intellectual functioning was slightly below average and significantly lower compared to the control group. This result is congruent with the numerous studies showing a small downward shift in mean IQ scores, which are mostly found around the low average to average ranges [25]. It is however unlikely that these different levels of intellectual functioning can explain the discrepancy evidenced in our electrophysiological measures. Indeed, our correlational analysis revealed no association between SSVEP SNRs and IQ in our sample. A comparable result was found in another EEG study investigating visual processing in NF1 that showed no correlation between IQ scores and electrophysiological measures which, in their case, were the amplitude of the visually evoked potentials and the amplitude of alpha oscillations [76].

Interestingly, our SSVEP measures were significantly related with behavioral symptoms measured through parental questionnaires in children with NF1. In the control group, no correlation remained significant after correction for multiple comparisons. These findings mainly confirmed our hypothesis stating that lower SSVEP amplitude would be associated with increased symptomatology*.* In NF1 participants, smaller SSVEP responses at 10 Hz and 15 Hz were correlated with higher inattention symptoms as measured by the Conners 3. Smaller SSVEP responses at 15 Hz were also correlated with higher scores on the Conners 3 global scale of ADHD-related symptoms (e.g. distractibility, agitation, impulsivity, emotional lability). Endogenous attention is known to modulate SSVEP amplitude and phase coherence. In experimental protocols where two stimuli flickering at different frequencies are presented simultaneously, the shift of attention towards one stimulus was shown to enhance the power of the SSVEP generated by the attended stimulus [64, 77–79]. In our study, different stimulation frequencies were presented sequentially, rather than simultaneously, which did not require participants to voluntarily shift their attention during the task. However, the optimal processing of the different colored icons presented at varying frequencies requires effective adaptation of the neural population’s activity and synchronization, which appears to be more affected in children who show increased attentional problems in daily life.

In children with NF1, the SSVEP response resulting from the 15 Hz stimulation was also negatively related with symptoms of emotional/behavioral problems measured by the CBCL questionnaire and this correlation survived correction for multiple comparisons. The CBCL Total problems scale combines symptoms of internalizing and externalizing problems, as well as symptoms of attention, social, and thought problems. In a study investigating emotional and behavioral problems in a large sample of children and adolescents with NF1 (*N* = 183), a mean score of 58.3 (± 10.3) on the CBCL Total problems was reported, which is in line with our results indicating a mean score of 57.3 (± 11.2) [80]. Interestingly, in another study using the same questionnaire, these emotional/behavioral problems were found to be significantly increased in children presenting with the NF1-ADHD comorbidity when compared to NF1 children without ADHD [81]. Our results thus suggest that the neural response to the 15 Hz stimulation covaries with a wide range of emotional/behavioral difficulties in children with NF1, which in turn might be related to the severity of ADHD symptomatology.

Finally, no correlation was found between the EEG measures and ASD-related symptoms in our sample. The severity of the ASD symptomatology in our NF1 group is consistent with most of the previous findings in the literature. In a population-based study of over 100 children with NF1 aged from 4 to 16 years, the mean total score reported on the SRS was between the high average and superior to average ranges (*T* score around 63) [82], while a mean score in the high average range (*T* score = 58.5) was found in our study. Given the sensory processing abnormalities and GABAergic dysfunction also evidenced in ASD, the integrity of the steady-state response has been studied in this population. Two studies have reported reduced SSVEP amplitudes in the occipital region of children with ASD, one regarding the SSVEP first harmonic [83] and the other, at the second harmonic [53]. However, further investigation is needed to determine whether these markers of sensory processing alterations vary with the severity of ASD symptoms in NF1 [84].

### Impact of psychostimulant medication

Studies have identified neurochemical alterations underlying the attentional system dysfunction in mouse models of NF1. Genetically engineered mouse models with a heterozygous knockout mutation of the neurofibromatosis gene (Nf1+/−) have shown reduced expression of the Nf1 gene and have been developed to study various aspects of the NF1 phenotype [85]. In specific types of Nf1 +/− mice, decreased exploratory and attentional behaviors were found to be a consequence of reduced striatal dopamine, and both the neurochemical and behavioral deficits were reversed by treatment with methylphenidate [8, 86]. In children with NF1, treatment with methylphenidate was shown to improve performance on a computerized attention task [81] and to reduce parent-reported ADHD symptoms [23]. However, the impact of psychostimulant medication on markers of brain activity and sensory processing remains unknown in NF1. In our study, we explored how the psychostimulant medication, taken by a subset of the NF1 group (*N* = 8), would affect the steady-state response.

Our results showed a significant increase in the SSVEP response generated by the 6 Hz stimulation with the intake of psychostimulant medication. This finding partially confirmed our hypothesis stating that the intake of psychostimulant would normalize the EEG signal since an improvement in SSVEP amplitude was found at the lowest stimulation frequency, but not at the higher frequencies, which seems to be more significantly impaired in the NF1 group. The absence of significant impact on the SNRs of the 10 Hz and 15 Hz SSVEPs could indicate that synchronization of neural activity at higher frequencies is more severely affected by the imbalances in neurotransmission found in NF1 and thus, less easily restored with psychostimulant medication. Interestingly, the 6 Hz SSVEP was also the only experimental condition where no correlation was found with any of the behavioral symptoms, which supports the idea that the neural responses to higher frequency stimulation are more closely related to the severity of the phenotype. It is also possible that the neural response to the 6 Hz stimulation is more sensitive to top-down attentional modulation and thus, more reactive to the intake of psychostimulant medication that strengthens the connectivity of the attentional networks [54]. Although further experiments are needed to replicate our findings regarding the impact of psychostimulant medication, our results suggest that the SSVEP response could potentially serve as a relevant marker for therapeutic interventions in NF1. Future studies could determine if the increased SSVEP response is associated with cognitive and behavioral improvements following treatment with psychostimulant medication.

## Limitations and perspectives

Limitations of this study include the relatively small sample size, especially for the investigation of the simulant medication’s impact on the EEG measures. More participants would be needed to increase the power of our analysis and robustly confirm the absence of effect from medication intake at higher frequencies. More participants would also be needed to determine if different types of medication (e.g., lisdexamfetamine vs methylphenidate) induce dissociable effects on the EEG measures. Furthermore, the absence of a group of non-NF1 participants with ADHD limits our ability to disentangle the implication of the NF1 and ADHD diagnoses in the altered SSVEP response evidenced in this study. Therefore, adding a group of participants with a diagnosis of ADHD only would be relevant to determine if their electrophysiological profile is dissociable from that of children with the NF1-ADHD comorbidity, and if the associations found between SSVEP responses and inattention symptoms are specific to the NF1 diagnosis. Also, our control group’s mean IQ score was found in the upper limit of the average range and was significantly higher than the NF1 group’s mean IQ. Considering that no correlation was found between SSVEP responses and IQ, it is unlikely that this discrepancy in intellectual functioning could explain our group differences in terms of EEG signal. However, to confirm that the altered processing of rhythmic visual stimuli is characteristic of NF1 (or of the NF1-ADHD comorbidity) regardless of IQ, it may be interesting to compare NF1 and controls’ SSVEP responses with IQ matched samples. Another limit of the study is the absence of neuropsychological tests assessing the participants’ visual attention abilities. We have shown that children’s neural response was related to the severity of inattention symptoms exhibited in daily life, but it would be relevant to see if it also covaries with their performance on cognitive tests of attentional skills. Moreover, it would be interesting to see how the modulation of attention during the visual stimulation, with a Posner cueing paradigm for example, affects the steady-state response in children with NF1. A passive task with attractive visual stimuli, as we used in our study, is however well suited for young children and allowed us to obtain quality recordings with our participants as young as 4 years old. It bears repeating that no significant difference was found between groups in terms of pre-processing indicators (number of ICA components and epochs rejected). Therefore, group differences in SSVEP responses can not be explained by reduced quality of the EEG recordings in one group or the other.

## Conclusions

In conclusion, visual processing abnormalities were identified in children with NF1 using SSVEP measures. The reduced SSVEP responses found in NF1 suggest decreased synchronization in the activity of neuronal populations in the visual cortex, which could be a consequence of neurochemical dysfunction, notably in the GABAergic system, and structural abnormalities in the visual cortex and thalamus. Our EEG measures seemed to be correlated with ADHD-related symptoms as well as with emotional/behavioral problems exhibited by children with NF1 in daily life. Moreover, the intake of psychostimulant medication in a subset of the NF1 group improved the SSVEP response resulting from the visual stimulation at the lowest frequency (i.e., 6 Hz). Taken together, these findings indicate that SSVEP measures can potentially be sensitive EEG biomarkers and be used in translational studies or clinical trials aimed at restoring alterations in brain activity resulting from pathogenic variants in the NF1 gene.

## Supplementary Information

**Additional file 1.** Inclusion of participants with optic pathway gliomas in the EEG analyses.

**Additional file 2.** Coloured icons presented at 6Hz, 10 Hz or 15 Hz during the visual task.

## Data Availability

NF1 participants’ EEG data is available in the Additional file 1. Other data used and analyzed during the current study are available from the corresponding author on reasonable request.

## Abbreviations

NF1: Neurofibromatosis type 1
Nf1 +/−: Heterozygous knockout mutation of the neurofibromatosis gene (Nf1+/−)
ADHD: Attention deficit hyperactivity disorder
ASD: Autism spectrum disorder
EEG: Electroencephalography
fMRI: Functional magnetic resonance imaging
MEG: Magnetoencephalography
PET: Positron emission tomography
GABA: Gamma-aminobutyric acid
SSVEP: Steady-state visual evoked potential
SNR: Signal-to-noise ratio
Hz: Hertz
FFT: Fast Fourier Transform
Conners 3-P: Conners 3rd Edition–Parent
SRS-2: Social responsiveness scale, 2nd edition
CBCL: Child behavior checklist
IQ: Intellectual quotient
FDR: False discovery rate
