# Supplementary material for: Steady-state visual evoked potentials in children with neurofibromatosis type 1: associations with behavioral rating scales and impact of psychostimulant medication
Source: J Neurodev Disord. 2022 Jul 22;14:42. doi: 10.1186/s11689-022-09452-y (PMC9306184; doi:10.1186/s11689-022-09452-y)
Supplement: Supplementary file 1 — Additional file 1. Inclusion of participants with optic pathway gliomas in the EEG analyses. [file 11689_2022_9452_MOESM1_ESM.docx]

Additional file 1. Inclusion of participants with optic pathway gliomas in the EEG analyses.

The non-parametric Mann-Whitney test was used to ensure that the EEG measures of the three NF1 participants with optic pathway gliomas did not differ from the rest of the group and could be included in the analyses. SNRs of the SSVEP responses were not significantly different when compared between participants with optic pathway gliomas (Mdn = 3.41, 3.07, 3.34 for the 6 Hz, 10 Hz, 15 Hz stimulation) and the rest of the NF1 group (Mdn = 4.52, 4.67, 4.53 for the 6 Hz, 10 Hz, 15 Hz stimulation) for either the 6 Hz stimulation (*U* = 31.00, *p* = 0.67), the 10 Hz stimulation (*U* = 18.00, *p* = 0.17) or the 15 Hz stimulation (*U* = 24.00, *p* =0.35). To support the conclusion that these three participants with optic pathway gliomas can be included in the analyses, a table showing where the SNR measures of these participants lie in the NF1 distributions is provided below.

Rank of the SNR measures of participants with optic gliomas in the NF1 distributions for each stimulation frequency

| **SNR at 6 Hz (dB)** | **SNR at 10 Hz (dB)** | **SNR at 15 Hz (dB)** |
| --- | --- | --- |
| 0,36 | 0,47 | 0,26 |
| 0,87 | 0,52 | 0,72 |
| 0,88 | 0,84 | 1,08 |
| 1,71 | 1,18 | 1,58 |
| 2,71 | 1,25 | 2,49 |
| 2,72 | 1,93 | 2,51 |
| 2,94 | 1,97 | 2,60 |
| 2,95 | 2,47 | 3,34 |
| 2,98 | 2,59 | 3,42 |
| 3,39 | 2,70 | 3,61 |
| 3,41 | 3,07 | 3,86 |
| 3,80 | 3,92 | 4,06 |
| 3,83 | 4,30 | 4,20 |
| 4,52 | 4,56 | 4,50 |
| 4,52 | 4,60 | 4,53 |
| 4,58 | 4,66 | 4,87 |
| 4,61 | 4,80 | 4,99 |
| 4,62 | 5,19 | 5,11 |
| 4,88 | 5,43 | 5,13 |
| 5,08 | 5,45 | 5,23 |
| 5,17 | 5,56 | 5,43 |
| 5,32 | 6,52 | 6,30 |
| 5,45 | 6,54 | 7,76 |
| 6,89 | 6,60 | 8,03 |
| 7,47 | 6,96 | 8,16 |
| 7,57 | 7,12 | 8,23 |
| 7,71 | 9,19 | 9,91 |
| 8,54 | 9,30 | 10,41 |

*Note. The SNR measures of participants with optic pathway gliomas are shown in red.*
