# Supplementary figures and images for: Steady-state visual evoked potentials in children with neurofibromatosis type 1: associations with behavioral rating scales and impact of psychostimulant medication
Source: J Neurodev Disord. 2022 Jul 22;14:42. doi: 10.1186/s11689-022-09452-y (PMC9306184; doi:10.1186/s11689-022-09452-y)

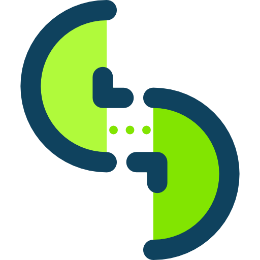

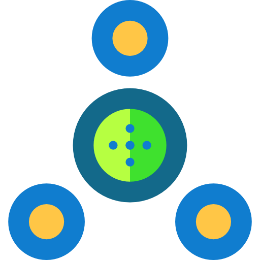

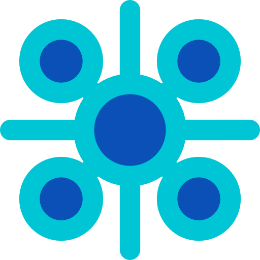

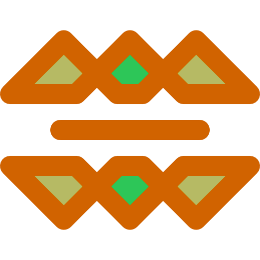

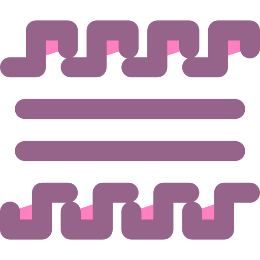

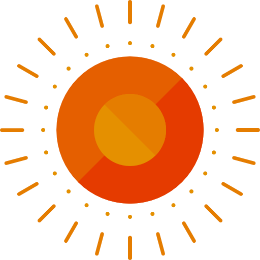

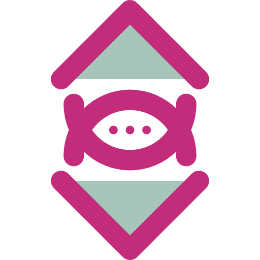

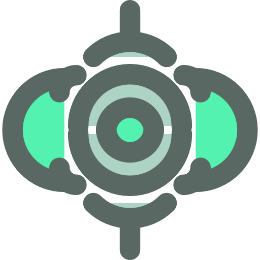

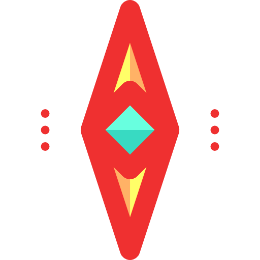

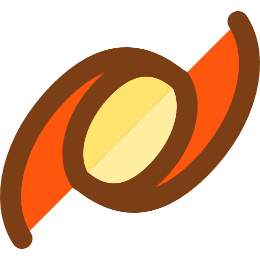

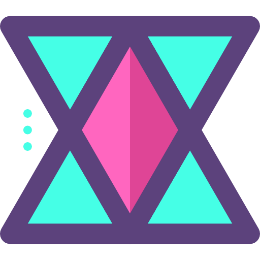

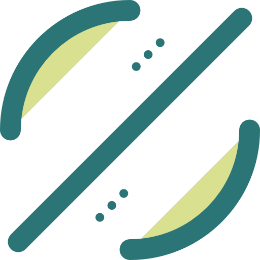

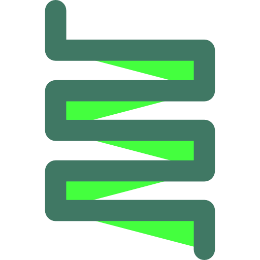

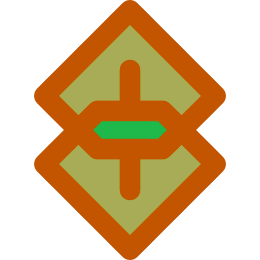

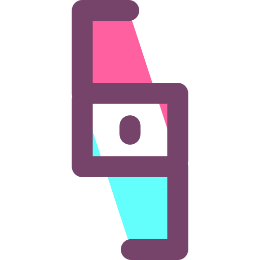

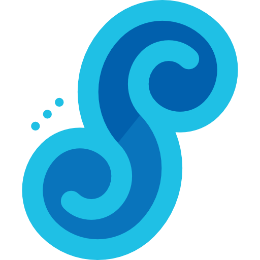

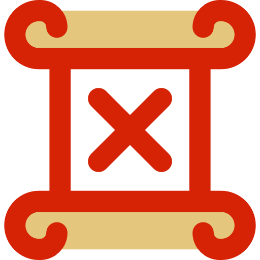

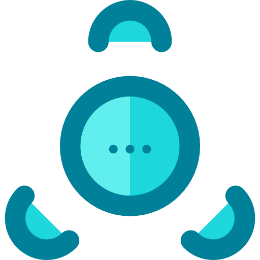
 Additional file 2. Coloured icons presented at 6Hz, 10 Hz or 15 Hz during the visual task.

Supplement: Supplementary file 2 — Additional file 2. Coloured icons presented at 6Hz, 10 Hz or 15 Hz during the visual task. [file 11689_2022_9452_MOESM2_ESM.docx]
